# A Sample of Female Adolescent Self-Identified Vegetarians in New Zealand Consume Less Protein and Saturated Fat, but More Fiber than Their Omnivorous Peers

**DOI:** 10.3390/nu14030711

**Published:** 2022-02-08

**Authors:** Meredith Peddie, Tessa Scott, Chaya Ranasinghe, Elizabeth Fleming, Kirsten Webster, Rachel Brown, Lisa Houghton, Jillian Haszard

**Affiliations:** Department of Human Nutrition, University of Otago, Dunedin 9016, New Zealand; tessa.scott@otago.ac.nz (T.S.); chaya.ranasinghe@otago.ac.nz (C.R.); liz.fleming@otago.ac.nz (E.F.); kirsten.webster@otago.ac.nz (K.W.); rachel.brown@otago.ac.nz (R.B.); lisa.houghton@otago.ac.nz (L.H.); jill.haszard@otago.ac.nz (J.H.)

**Keywords:** adolescence, fat carbohydrate, protein, food groups

## Abstract

This study aimed to describe the intake and food sources of macronutrients in vegetarian and non-vegetarian adolescent females. Cross-sectional data was collected between February and September 2019. Adolescent females, aged 15 to 18 years old, were recruited throughout New Zealand. Intakes were assessed via two 24-h diet recalls, adjusted to represent usual intake using the multiple source method. Of the 254 participants, 38 self-identified as vegetarian. Vegetarians had similar carbohydrate and fat intakes compared to non-vegetarians; however, their protein intakes were 2.1% kJ lower (95% confidence interval (CI) −3.0 to −1.1%). Vegetarians also consumed 1.1% kJ less saturated fat (95% CI –2.1 to −0.1%), 1.3% kJ (95% CI 0.7 to 1.9) more polyunsaturated fat, and 5 g/day (95% CI 1.8 to 8.0) more fiber than non-vegetarians. When consumed, bread-based dishes and discretionary foods were the highest sources of energy, fat, and carbohydrate in both vegetarians and non-vegetarians. This suggests that some adolescents, including vegetarians, were obtaining high amounts of fat and carbohydrate from food groups associated with poorer dietary quality. We recommend further research to assess how the changing food environment is influencing vegetarian eating patterns and their associations with health outcomes in the wider population.

## 1. Introduction

Vegetarians—those who follow a diet that excludes meat, poultry or seafood, but include dairy and eggs—are more likely to have a lower body mass index (BMI), gain less weight throughout adulthood [1], and have lower cholesterol concentrations [1] when compared to non-vegetarians. Furthermore, vegetarian diets have been associated with lower risk of some cancers [1] and up to 24% lower risk of ischemic heart disease in adults [1]. Also, a higher plant-based eating score is associated with a 14% lower risk of coronary heart disease [2]. This lower risk can, at least in part, be attributed to the fact that many vegetarians have lower energy, saturated fat and sodium intakes, while consuming more polyunsaturated fat, fruits, and vegetables [3,4,5].

Much of the data that underpins our understanding of how following a vegetarian diet may affect disease incidence was collected in the early 2000s, or even earlier [6,7,8]. Recent advances in food technology and fortification practices have led to widespread availability of commercially-produced plant-based substitutes for meat and dairy products [5]. These changes to the food supply may have changed the foods, and thus macronutrients, consumed by modern vegetarians, potentially influencing diet quality.

The quality of a vegetarian diet may be even more important during adolescence. Adolescence is a time of increased energy and nutrient requirements due to accelerated growth and development [9] and, therefore, achieving an adequate and well-balanced diet is of increased importance. Dietary patterns developed over this time are also likely to be maintained through adulthood [10], and recent research indicates that young adults who follow a high-quality plant-based diet experience ~50% lower risk of developing cardiovascular disease in later adulthood [11]. Similar to research in adults, much of the evidence on the eating patterns of vegetarian adolescents is close to 20 years old [12,13,14,15]. This research indicates that vegetarian adolescents also tended to have lower total and saturated fat intakes (3–8% and 2–13% of total energy lower, respectively) [12,14,15], lower protein intakes (0.6 to 4% of total energy lower) [12,13,14,15], higher carbohydrate intakes (4–10% of total energy higher) [12,14,15], and higher polyunsaturated fat intakes (0.3–3% of total energy higher) [14,15]. The most recent evidence from the United States (US) indicates that vegetarians still have lower total and saturated fat intakes (3% total energy lower for both) and higher carbohydrate (5% total energy higher) and polyunsaturated fat intakes (1% total energy higher), but no evidence of differences in protein intakes [16]. However, this study was done among two Seventh Day Adventist populations in the US, where both vegetarians and non-vegetarians were found to have healthier diets (fruit and vegetable intake ~140–300% higher) than those recorded among adolescents who participated in National Health and Nutrition Examination Survey (NHANES) 2011–2012 [16].

Given the increasing popularity of plant-based eating in response to sustainability and environmental concerns, as well as widely disseminated health reports (such as EAT-Lancet [17]), understanding the nutritional consequences of the modern vegetarian diet in young women is imperative. This is even more relevant given the changing food environment where processed foods are cheap and readily available. Our current understanding of macronutrient intakes and food groups that contribute to these in vegetarian adolescents is limited and outdated. Therefore, the aim of this study was to describe the energy and macronutrient intakes of vegetarian and non-vegetarian adolescent females, and to compare and contrast the main food sources of these nutrients.

## 2. Materials and Methods

### 2.1. Study Design

The Survey of Nutrition, Dietary Assessment and Lifestyles (SuNDiAL) Project was a cross-sectional survey of female adolescents aged 15–18 years, conducted throughout New Zealand in 2019. A sample size of 300 students was required to detect a 0.5 standard deviation difference in nutrient intakes between vegetarians and non-vegetarians, given an estimated 20% prevalence of vegetarians. A detailed summary of the methods is presented elsewhere [18]. The study was approved by the University of Otago Human Ethics Committee (Health): H19/004 and is registered with the Australian New Zealand Clinical Trials Registry: ACTRN12619000290190.

### 2.2. Participants

Initially, high schools in eight predetermined locations throughout New Zealand were invited to participate. These locations (Dunedin, Christchurch, Nelson, Wellington, New Plymouth, Whangarei and Wanaka) were conveniently chosen based on the locations of data collectors. From the eight locations, 13 schools agreed to participate. Adolescent girls were then recruited from within each of these schools by dissemination of study information via presentations to the whole school, year groups, or individual classes (depending on school preference). Recruitment (and data collection) occurred at two timepoints: from February to April 2019, and July to September 2019. Adolescents who self-identified as female, were aged between 15 and 18 years, could speak and understand English, and were not pregnant were eligible to participate. Online informed consent was obtained from all participants, and from the parent or guardian of participants aged under 16 years.

During the second phase of data collection, it became apparent that the prevalence of vegetarians in the recruited sample was lower than expected. Therefore, targeted recruitment of vegetarians was carried out both within the recruited schools, and from advertisements in the general public (provided they met the inclusion criteria) in Dunedin and Christchurch. When participants were recruited from the general public, data collection occurred at research clinics in Dunedin and Christchurch instead of in schools.

### 2.3. Demographics

Prior to in-school data collection, participants provided consent and completed an online Research Electronic Data Capture (REDCap, Version 10, Vanderbilt University, Nashville, TN, USA) [19] questionnaire. This questionnaire included basic demographic and health questions, but also asked participants “Are you vegetarian or vegan?”. If the participant answered in the affirmative, they were considered vegetarian in the analysis presented here. Ethnicity was self-identified and then prioritised into four ethnic groups using the following order: Māori, Pacific, Asian, New Zealand European, and other. Socio-economic status was determined based on the geographical mesh-block of the participant’s residential address. Each mesh-block in New Zealand is categorised into deciles of New Zealand Index of Deprivation (2018). Deciles were then collapsed into 3 categories representing low (deciles 1–3) moderate (deciles 4–7) and high (deciles 8–10) deprivation.

### 2.4. Demographics

At the in-school data collection session weight (measured using one of Medisana PS420, Nuess, Germany; Salter 9037 NK3R, Kent, United Kingdom; Seca Alpha 770, Hamburg, Germany; or Soehnle Style Sense Comfort 400 scales, Backnang, Germany) and height (measured using a Seca 213, Hamburg, Germany or Wedderburn stadiometer, Sydney, Australia) were measured in duplicate with the participant wearing no shoes and light clothing, to the nearest 0.1 kg or cm, respectively. A third measurement was taken if the first two measures differed by more than 0.5 units, and the mean of the closest two measurements used as the ‘true’ value. Body mass index was calculated by weight (in kg) divided by height (in m) squared. Body mass index z-scores for age and height were used to classify participants as healthy weight, overweight or obese using the World Health Organization (WHO) child growth standards [20].

### 2.5. Usual Dietary Intake

Dietary intake was assessed using two 24-h diet recalls. The first recall was conducted at the in-school data collection session and the second was completed over a voice or video call on a non-consecutive day following this. Both recalls were performed using a multiple-pass technique (for more information see [18]). All 24-h diet recalls were entered into FoodWorks dietary analysis software (Version 9, Xyris Software Ltd., Brisbane, Australia) using Foodfiles 2016 (New Zealand Institute for Plant and Food Research Ltd. (Auckland, New Zealand) and the New Zealand Ministry of Health) and nutrient data from commonly consumed recipes collated as part of the 2008/09 New Zealand Adult Nutrition Survey [21]. Intakes of each macronutrient were adjusted to represent usual intakes based on the estimated within-person variance of vegetarians and non-vegetarians using the multiple source method [22].

### 2.6. Food Groups

All food items reported in the 24-h diet recall were allocated in the food group categories used in the 2008/09 New Zealand Adult Nutrition Survey [21]. These 24 food groups were further amalgamated into 25 food groups in total: ‘Biscuits’, ‘Cakes & muffins’, ‘Puddings & desserts’, ‘Sugar & sweets’, ‘Snack foods’, and ‘Snack bars’ were combined into a ‘Discretionary food’ group; ‘Beef & veal’, ‘Lamb & mutton’, ‘Pork’, and ‘Other meat’ were combined into a ‘Red meat’ group (with bacon, ham and corned beef being moved to the Processed meat food group; ‘Soups & stocks’ and ‘Savoury sauces & condiments’ were combined into a ‘Soups, sauces, & condiments’ group; and ‘Fats & oils’ were combined with ‘Butter & margarine’ in a ‘Fats’ group. Additionally, new categories of ‘Vegetarian meat alternatives’ and ‘Legumes’ were created (see Supplementary Materials, Table S1).

### 2.7. Statistical Analysis

All statistical analysis was carried out in Stata 17.0 (StataCorp, College Station, TX, USA). Differences in demographics between vegetarians and non-vegetarians were described and assessed using a two-tailed, unpaired *t*-test (age and BMI z-score) or a Fisher’s exact test (ethnicity, household-level deprivation, and weight status). Means and standard deviations for nutrient intakes were calculated for each group (vegetarian and non-vegetarian) and mean difference, 95% confidence intervals (CI), and *p*-values estimated using a *t*-test. Estimates adjusted for age and energy intake were also calculated using a linear regression model with robust standard errors.

Macronutrient intakes as a percent of energy were compared to the Acceptable Macronutrient Distribution Range (AMDR) [23] and proportions of each group above and below these ranges were reported. To describe the food group sources of each macronutrient, initially the proportion of the groups who consumed each food group was reported (as the number of consumers illustrates how much this food group contributes to intake of the whole group). Differences between the vegetarian and non-vegetarian groups were tested with a Fisher’s exact test. Subsequently, the median and 25th and 75th percentiles of nutrient intake for the participants who consumed the food group was determined (those who did not consume the food group had zero nutrient intake from that food group). This illustrates, on average, how much of the nutrient the participants were getting from this food group if they consumed it. Differences between vegetarians and non-vegetarians were assessed with a Mann-Whitney test.

Residuals of regression models were plotted and assessed for homogeneity of variance and normality. No adjustment was made for multiplicity. Statistical significance was set at *p* < 0.05.

## 3. Results

### 3.1. Demographics

Five hundred and fourteen students responded to the recruitment drive, with 291 consenting and enrolling to participate. A total of 254 participants completed both the online questionnaire, provided anthropometric measurements, and completed at least one 24-h recall. Of the 38 who self-reported being vegetarian, nine reported being vegan, one reported eating meat occasionally, three reported eating chicken or poultry, nine reported that they ate fish or seafood and 28 reported that they ate eggs. On average the vegetarians were older and had a lower BMI z-score than their non-vegetarian counterparts (Table 1).

### 3.2. Energy and Macronutrient Intakes

Although not statistically significant, energy intakes were lower in vegetarians by as much as 500 kJ/day. Total carbohydrate intakes were similar between vegetarians and non-vegetarians (Table 2). Vegetarian protein intakes were 9 g/day (95% CI −12.7 to −5.3 g/day) or 2.1% of energy (95% CI −3.0 to −1.1) lower than non-vegetarians (Table 2). Correspondingly, a higher percentage of vegetarians (73.7% compared to 43.1%) were below New Zealand AMDR for protein, when compared to non-vegetarians (Supplementary Materials, Table S2). Four vegetarians reported consuming either poultry or seafood during their recalls, excluding these participants on the basis that they are not truly vegetarian lowered the mean (standard deviation, SD) protein intake for vegetarians slightly to 59.7 (17.3) g/day. Vegan participants did not have noticeably lower mean (SD) protein intakes (65.0 (16.4) g/day) than non-vegans. The majority of protein intakes for both vegetarians and non-vegetarians were above the estimated average requirement (EAR) of 35 g/day, although slightly more non-vegetarians were achieving the EAR when compared to vegetarians (99.1% vs. 94.7% respectively; *p* = 0.048) (Supplementary Materials, Table S2).

While total fat intake did not differ markedly between vegetarians and non-vegetarians, most of the participants in both groups had intakes above the AMDR (percentage above the AMDR = 71.1% and 73.6%, respectively).

The composition of fat did differ, with vegetarians consuming 2.7 g/day (95% CI 1.4 to 4.0 g/day), or 1.3% of energy (95% CI 0.7 to 1.9%) more polyunsaturated fat and 2.2 g/day (95% CI −4.6 to 0.1 g/day) or 1.1% of energy (95% CI −2.1 to −0.1%) less saturated fat than non-vegetarians. Additionally, a higher percentage of vegetarians, compared to non-vegetarians, had saturated fat intakes below 10% of energy intake (23.7% vs. 6%; *p* < 0.001). Monounsaturated fat intakes were not markedly different between vegetarians and non-vegetarians (Table 2). Vegetarians also consumed on average, 5 g/day (95% CI 1.8 to 8.0 g/day) more fiber a day than their non-vegetarian peers. However, sugar intakes were not significantly different between the groups (difference 0.0% kJ; 95% CI −2.1 to 2.05 kJ).

### 3.3. Food Groups

The consumption of food groups differed between vegetarians and non-vegetarians (Table 3). Participants consumed a mean of 12 different food groups on their recall days (ranging from 4 to 18 food groups, out of a possible 25). A higher percentage of non-vegetarians were consumers of animal-based food products such as Eggs & egg-based dishes (27% vs. 8%), Red meat (38% vs. 0%), Poultry (54% vs. 3%), Processed meat (44% vs. 0%), and Pies & pasties (15% vs. 3%), while a higher percentage of vegetarians were consumers of Vegetarian meat alternatives than non-vegetarians (32% vs. 1%). While the percentage of vegetarians and non-vegetarians consuming these food groups differed, when these food groups were consumed by both groups, the amount of energy, carbohydrate, fat, and protein provided by food groups did differ significantly (Table 3).

### 3.4. Contribution of Food Groups to Macronutrient Intake

In vegetarians the five food groups that contributed the largest amounts of energy (when consumed) were Bread-based dishes, Discretionary foods (snacks and desserts), Vegetarian meat alternatives (e.g., vegan burgers, vegan sausages, tofu products) Pies & Pasties, and Grains & Pasta (Table 4). In non-vegetarians the top five contributors to energy were Bread-based dishes, Pies & Pasties, Discretionary foods, Grains & Pasta and Bread. A similar number of vegetarians and non-vegetarians reported Legumes and Vegetables. However, while the overall contribution to energy from these food groups was small, both Legumes and Vegetables contributed a significantly larger amount of energy to the diets of vegetarians who consumed them when compared to non-vegetarians. 

In vegetarians, the five food groups that contributed the largest amounts of total fat (when consumed) were Bread-based dishes, Discretionary foods, Vegetarian meat alternatives, Poultry (consumed by only one participant) and Pies & pasties (Supplementary Materials, Table S3). In non-vegetarians, the top five contributors to total fat intake were Pies & pasties, Bread-based dishes, Discretionary foods, Red meat and Sausages & processed meat. Similar numbers of vegetarians and non-vegetarians reported consuming Vegetables and Legumes; however, while the contribution to fat intake from these food groups was small, both contributed a larger amount of fat to the diets of vegetarians who consumed them compared to non-vegetarians. The specific vegetable dishes that contributed to this higher amount of fat included salads, stuffed eggplant, and stir-fry. For Legumes, the foods included refried beans, hummus, falafel, and bean-based burger patties.

Among vegetarians the food groups that contributed the most polyunsaturated fat to the diets of consumers were Vegetarian meat alternatives, Fish & seafood, Discretionary foods, Nuts & seeds, and Pies & pastries (Supplementary Materials, Table S4). Among non-vegetarians the food groups that contributed the most polyunsaturated fat to the diets of consumers were Nuts & seeds, Bread based dishes, Discretionary foods, and Poultry, with Fish & seafood and Pies & pastries, both providing, on average, 1.1 g/day of polyunsaturated fat a day when they were consumed. While similar numbers of vegetarians and non-vegetarians consumed Discretionary foods, Dairy products, and Milk, the amount of polyunsaturated fat these food groups provided were statistically different. However, the magnitude of these differences means they are unlikely to be meaningful. 

Among vegetarians, the food groups that contributed the most saturated fat to the diets of consumers were Bread-based dishes, Poultry (only consumed by one participant), Cheese, Dairy products and Discretionary foods (Supplementary Materials, Table S5). Among non-vegetarians, the food groups that contributed the most saturated fat to the diets of consumers were Pies & pasties, Bread-based dishes, Discretionary foods, Cheese, and Red meat. While similar numbers of vegetarians and non-vegetarians consumed Vegetables, Legumes, and Milk, the amount of saturated fat these food groups provided were statistically different. However, the magnitude of these differences means they are unlikely to be meaningful.

Among vegetarians, the food groups that contributed the most monounsaturated fat to the diets of consumers were Bread-based dishes, Discretionary foods, Poultry (only consumed by one participant), Vegetarian meat alternatives, and Pies & pasties (Supplementary Materials, Table S6). Among non-vegetarians, the food groups that contributed the most monounsaturated fat to the diets of consumers were Bread-based dishes, Pies & pasties, Nuts & seeds, Discretionary foods, and Red meat. While similar numbers of vegetarians and non-vegetarians consumed Vegetables and Legumes, the amount of monounsaturated fat these food groups provided were statistically different. However, the magnitude of these differences means they are unlikely to be meaningful.

The top five food group contributors to protein in vegetarians when consumed were Supplements containing energy (e.g., protein powder), Fish & seafood, Vegetarian meat alternatives, Bread-based dishes, and Eggs & egg-based dishes (Table 5). While these five food groups were rich sources of protein for vegetarians, they were not commonly consumed. For example, only 5.3% (*n* = 2) vegetarians reported consuming supplements containing energy, and only 7.9% (*n* = 3) vegetarians reported eating both Fish & seafood and Eggs & egg-based dishes. In non-vegetarians, the top five food group contributors to protein were Bread-based dishes, Red meat, Poultry, Fish & seafood, and Pies & Pasties. While similar numbers of vegetarians and non-vegetarians reported consuming Vegetables, Legumes, and Soups, sauces & condiments, all three of these food groups contributed a significantly larger amount of protein to the diet of vegetarians compared to non-vegetarians.

The top contributors to carbohydrate in vegetarians, when consumed, were Discretionary foods, Grains & pasta, Vegetarian meat alternatives, Pies & pasties, and Bread. In non-vegetarians, the top five contributors to carbohydrate intake were Bread-based dishes, Discretionary foods, Bread, Grains & pasta and Pies & Pasties. (Supplementary Materials, Table S7). While similar numbers of vegetarians and non-vegetarians reported consuming Vegetables and Soups, sauces & condiments, these two food groups contributed a significantly larger amount of carbohydrate to the diet of vegetarians compared to non-vegetarians. The top five contributors to fiber intake in vegetarians were Vegetarian meat alternatives, Legumes, Bread-based dishes, Vegetables, and Bread. In non-vegetarians the top four contributors to fiber intake, when consumed, were Vegetarian meat alternatives (although this was only consumed by one participant), Fruit, Bread-based dishes, and Bread, with Potatoes, kumara & taro and Breakfast cereals both providing 3.4 g fiber/day (Supplementary Materials, Table S8). While Grains & pasta were consumed by a similar number of vegetarian and non-vegetarians, when consumed, Grains & pasta contributed significantly more fiber to the intake of vegetarians compared to non-vegetarians.

## 4. Discussion

Despite the apparent increasing popularity of vegetarian diets, this study is one of few (especially in a non-seventh day Adventist population) to compare and contrast the macronutrient intakes of vegetarian and non-vegetarian adolescent females. As one might expect, fewer vegetarian adolescents who participated in this study reported eating animal-based food groups when compared to their non-vegetarian counterparts. However, when these food groups were consumed by vegetarians, their contribution to the macronutrient intake was similar to that of non-vegetarians. In fact, there were very few marked differences in the median intakes of macronutrients provided by different food groups when they were consumed, with the only exceptions being Vegetables, Legumes, and Soups, sauces & condiments, which provided more energy, fat, carbohydrate, and protein for vegetarians compared to non-vegetarians. Of note, however, is that despite these differences, these food groups are not highly ranked contributors to any of these nutrients.

It is then perhaps less surprising that in total, energy, fat, and carbohydrate intakes were similar between vegetarians and non-vegetarians—which means we accepted our hypothesis. This result, however, contrasts the results of previous studies which often indicate that the carbohydrate intakes are higher in vegetarians [12,14,15]. Differences in intakes between the current study and previous studies may, at least in part, be explained by changes in overall dietary patterns. In recent years there has been an increase in the popularity of higher fat, lower carbohydrate diets [24]. This is reflected in the high percentage of both groups whose fat intake falls above the AMDR, as well as the higher percentage of energy coming from fat when compared to the results for females of this age group from the 2008/09 New Zealand Adult Nutrition Survey (33.8%) [21]. Differences in the food groups contributing fat to the diet between the current study (Pies & pastries, Discretionary foods) and the 2008/09 New Zealand Adult Nutrition Survey (Bread-based dishes and Potatoes) also reflect a possible change away from carbohydrate-rich food staples. 

The higher fat intakes reported here, when compared to those reported more than 10 years ago [21], may be cause for concern. However, it appears that the higher total fat intakes are being driven by higher monounsaturated and polyunsaturated fat intakes instead of saturated fat. Total fat intakes were similar between vegetarians and non-vegetarians in the current study, but similar to previous research [12,14,15]. Vegetarians reported consuming more polyunsaturated fat (in this case ~3 g/day) and were more likely to be consuming less than 10% of energy from saturated fat. The more favourable fat composition may, at least in part, be being driven by the absence of red and processed meat in vegetarian diets. While only 26% of vegetarians reported consuming plant-based meat alternatives, they do appear to be a rich source of polyunsaturated fat. It also seems likely that larger servings, or more frequent consumption of vegetable and legume-based dishes are also contributing to the higher polyunsaturated fat intake of vegetarians. 

The fact that the protein intakes of vegetarians in this study were ~2% of total energy lower than their non-vegetarian peers is in line with older studies that report protein intakes that are between 0.6 and 4% of total energy lower [12,13,14,15]. It should be noted, however, that while protein intakes are lower, and many (75%) vegetarians reported protein intakes below the AMDR, the vast majority of both vegetarians and non-vegetarians in this study are achieving the EAR. Animal-based foods are often high in protein, and indeed this is illustrated in the food group analysis. Even though a small number of vegetarians report consuming foods such as supplements containing energy, fish & seafood, eggs & egg-based dishes, and poultry, when they were consumed, these foods ranked highly in terms of the amount of protein they contributed. This may suggest that strict vegetarians or vegans who are less likely to consume animal-based products may be even more at risk of lower protein intakes; however, this did not seem to be the case in the current analysis, although caution is required when interpreting this finding because of the low numbers of vegans (*n* = 9). Vegan participants did not have lower protein intakes than non-vegans. It is possible that vegans who have made a definitive choice to exclude a larger variety of foods from their diet are more careful at planning replacements than those who simply choose to exclude red meat.

Previous research comparing the diets of vegetarians and non-vegetarians found evidence that supports the idea that vegetarian diets tend to be of higher quality (e.g., higher fiber, polyunsaturated fat, and fruit and vegetable consumption) [14,16]. We also found some evidence to support this idea (higher fiber and polyunsaturated fat). However, the results also indicate that at least some of the vegetarians who participated in this study are getting a marked amount of fat and carbohydrate from Discretionary (or snack) foods. It is possible that the move away from the higher quality diet traditionally observed in vegetarians is the result of a broadening range of reasons for vegetarianism (i.e., those who choose to be a vegetarian for animal welfare reasons may have less interest in the overall quality of their diet than someone who chooses to be vegetarian because of the associated health benefits). It is also possible that the food industry has responded to the increasing popularity of vegetarianism and is now producing more snack foods that are “vegetarian friendly”. Interestingly, Vegetarian meat alternatives were consumed by only a third of vegetarians, but ranked highly in terms of the amount of energy, fat (5.8 g of polyunsaturated fat, 5.1 g of monounsaturated fat, and 3.8 g of saturated fat), protein, carbohydrate, and fiber they provided. The energy density of these foods should be given consideration, particularly given that plant-based foods are often seen as inherently healthier, and less energy dense options. It should also be noted that many of these participants will be living at home with their family and may be trying to follow a vegetarian diet in a household of non-vegetarians, with perhaps less autonomy over food shopping and preparation. This may lead them to exclude meat without careful substitution and instead increase intake of other, readily available non-meat foods. 

The data in this study was collected from adolescents across multiple locations throughout New Zealand; however, the results are limited by the fact that the sample is not nationally representative. Additionally, the prevalence of vegetarianism in the recruited sample was much lower than anticipated and highlights the need for representative data to be collected on rates of vegetarianism in New Zealand. Targeted recruitment of vegetarians was required to ensure sufficient numbers for comparison in the vegetarian group. This may mean that the final sample was underpowered to detect statistically significant differences and that bias may be present. Twenty-four-hour diet recalls are commonly used to assess diet in large studies; however, they, like any form of dietary assessment, are not free from bias. The careful description of the food sources of nutrient intake makes this a rich description of the diet and intakes of vegetarian and non-vegetarian adolescent females in New Zealand. 

Comparison of the food group results to other large surveys is limited because these studies only report nutrient intake from food groups using percent of total intake (for example see [21]). Presenting food groups as percentages of total intakes tends to be misleading because it does not provide any information about how much of the nutrient is consumed from different food groups (and therefore how much it contributes to intakes when it is consumed). It also does not provide any information about how many participants consumed the nutrient from each of the food groups on recall days. The inclusion of non-consumers and the use of relative measures (i.e., the percentage of a total intake of a nutrient that may vary dramatically between studies) makes the use of percent of total intakes to compare between studies (or even compare between groups within the same studies) inappropriate. Population estimates of intakes of nutrients from food groups have not been made here because they should not be made with data from 24-h recalls unless frequency of consumption is also measured (which was not done in the current study). Using 24-h recall data to produce population estimates is error-prone because much of the intake data is highly skewed, and does not recognise the fact that food group intakes vary widely from day-to-day, week-to-week, and season-to-season. Unless individuals are specifically avoiding a particular food group, most participants are likely to consume foods across the full range of food groups over the course of weeks or months. However, it is unlikely that a participant will report consuming foods from all food groups during a single, or even across two, 24-h recalls; indeed, the average number of food groups reported on recall days in this study was 12 out of the total of 25, with a maximum of 18. This highlights the fact that this data is unlikely to provide accurate estimates of food group intakes at a population level. 

## 5. Conclusions

There is very little evidence of inadequate macronutrient intakes in adolescent females. However, the higher fat intake, particularly saturated fat, of both vegetarians and non-vegetarians, when compared to this age group ~10 years ago is concerning and highlights the need for a more regular monitoring of the dietary intake of the population. Vegetarians consumed more fiber, more polyunsaturated fat, and less protein than their non-vegetarian peers. However, some vegetarians were obtaining a high proportion of their fat and carbohydrate intakes from food groups associated with poorer dietary quality. We recommend more regular monitoring of dietary intake of the New Zealand population, with a focus on populations who follow different dietary patterns such as vegetarianism. We also recommend longer term follow-up to reassess the established associations between vegetarian eating patterns and health outcomes given the possible shift in dietary patterns.

## Figures and Tables

**Table 1:** Demographic characteristics of self-identified vegetarians and non-vegetarians (*n* = 254).

	Non-Vegetarians (*n* = 216)	Self-Identified Vegetarians (*n* = 38)	*p*-Value
Age, mean (SD) years	16.8 (0.9)	17.1 (0.8)	0.009
Ethnicity, *n* (%)			0.557
NZEO ^a^	169 (78.2)	31 (81.6)	
Māori	32 (14.8)	7 (18.4)	
Pacific	6 (2.8)	0	
Asian	9 (4.2)	0	
Deprivation ^b^, *n* (%)			0.896
Low	83 (38.4)	16 (42.1)	
Medium	92 (42.6)	16 (42.1)	
High	41 (19.0)	6 (15.8)	
BMI z-score ^c^, mean (SD)	0.76 (0.97)	0.25 (0.81)	0.003
Weight status ^c^, *n* (%)			0.149
Healthy	138 (64.8)	31 (81.6)	
Overweight	51 (23.9)	5 (13.2)	
Obese	24 (11.3)	2 (5.3)	
Height, mean (SD) cm	166 (7)	166 (6)	0.912

^a^ NZEO: New Zealand European and other. ^b^ Deprivation is determined by the New Zealand (NZ) Deprivation Index (2018) with low: 1–3; medium: 4–7; and high: 8–10. ^c^ BMI z-scores determined using the World Health Organization (WHO) growth charts; *n* = 3 non-vegetarians were missing body mass index (BMI) z-score. Overweight was defined as BMI z-score ≥ 1 & < 2, with obese defined as BMI z-score ≥ 2. SD: standard deviation.

**Table 2:** Energy and macronutrient intakes in non-vegetarians and self-identified vegetarians (*n* = 254).

	Mean (SD) Daily Intake in Non-Vegetarians (*n* = 216)	Mean (SD) Daily Intake in Self-Identified Vegetarians (*n* = 38)	Mean Difference (95% CI) in Daily Intakes	*p*-Value	Adjusted ^a^ Mean Difference (95% CI) in Daily Intakes	*p*-Value
Energy, kJ	8023 (1913)	7519 (1664)	−504 (−1155, 146)	0.128	−442 (−1031, 147)	0.141
Fat, g/day	79.7 (23.6)	76.0 (19.9)	−3.7 (−11.7, 4.3)	0.366	1.6 (−2.4, 5.6)	0.430
Saturated fat, g/day	30.4 (9.5)	26.0 (9.3)	−4.5 (−7.7, −1.2)	0.008	−2.2 (−4.6, 0.1)	0.065
Monounsaturated fat, g/day	29.7 (9.3)	28.9 (7.5)	−0.8 (−3.9, 2.4)	0.627	0.9 (−0.8, 2.7)	0.301
Polyunsaturated fat, g/day	11.8 (5.1)	13.7 (5.1)	1.9 (0.1, 3.7)	0.034	2.7 (1.4, 4.0)	<0.001
Protein, g/day	74.6 (20.1)	61.3 (17.1)	−13.3 (−20.1, −6.5)	<0.001	−9.0 (−12.7, −5.3)	<0.001
Protein ^b^, g/kg/day	1.16 (0.36)	1.03 (0.32)	−0.13 (−0.25, −0.01)	0.037	−0.06 (−0.14, 0.02)	0.162
Carbohydrate, g/day	225 (60)	220 (55)	−6 (−26, 15)	0.589	8 (0, 17)	0.057
Sugars, g/day	100 (42)	92 (34)	−9 (−23, 5)	0.193	−2 (−11, 7)	0.646
Fibre, g/day	23.5 (8.8)	27.4 (11.6)	3.8 (0.6, 7.0)	0.019	5.0 (1.8, 8.1)	0.002
Fat, % kJ	37.3 (5.7)	38.2 (6.2)	0.9 (−1.1, 2.9)	0.369	1.0 (−1.0, 3.1)	0.320
Saturated fat, % kJ	14.3 (2.8)	13.0 (3.4)	−1.3 (−2.3, −0.3)	0.014	−1.1 (−2.1, −0.1)	0.035
Monounsaturated fat, % kJ	13.9 (2.6)	14.5 (2.6)	0.6 (−0.3, 1.5)	0.224	0.5 (−0.4, 1.4)	0.305
Polyunsaturated fat, % kJ	5.5 (1.6)	6.8 (1.8)	1.3 (0.7, 1.8)	<0.001	1.3 (0.7, 1.9)	<0.001
Protein, % kJ	15.6 (2.7)	13.6 (2.2)	−2.0 (−2.9, −1.1)	<0.001	−2.1 (−3.0, −1.1)	<0.001
Carbohydrate, % kJ	46.9 (5.9)	48.7 (5.4)	1.8 (−0.2, 3.8)	0.082	1.7 (−0.4, 3.8)	0.104
Sugars, % kJ	20.4 (5.8)	20.7 (6.1)	−0.3 (−2.4, 1.8)	0.806	0.0 (−2.1, 2.0)	0.973

^a^ Adjusted for age and energy intake. ^b^ Three non-vegetarians were missing weight data. CI: confidence interval.

**Table 3:** Consumers of food groups (*n* = 254).

Non-Vegetarians (*n* = 216)		Self-Identified Vegetarians (*n* = 38)	
Food Group	% of Group Who were Consumers	Food Group	% of Group Who were Consumers
Discretionary foods	94.4	Discretionary foods	86.8
Bread	82.4	Vegetables	86.8
Vegetables	80.6	Bread	76.3
Fruit	78.2	Fruit	73.7
Soups, sauces, & condiments	73.2	Soups, sauces, & condiments	65.8
Non-alcoholic beverages	72.7	Milk	63.2
Grains & pasta	66.7	Non-alcoholic beverages	63.2
Milk	65.7	Grains & pasta	60.5
Fats	63.0	Nuts & seeds	52.6
Potatoes, kumara, & taro	56.9	Potatoes, kumara, & taro	52.3
Poultry	53.7 ^a^	Fats	50.0
Cheese	49.1	Dairy products	44.7
Sausages & processed meat	44.4 ^a^	Cheese	42.1
Dairy products	43.5	Legumes	39.5
Breakfast cereals	43.5	Breakfast cereals	36.8
Nuts & seeds	39.8	Vegetarian meat alternatives	31.6 ^a^
Red meat	38.4 ^a^	Bread-based dishes	29.0
Bread-based dishes	31.0	Eggs & egg-based dishes	7.9 ^a^
Eggs & egg-based dishes	27.3 ^a^	Fish & seafood	7.9
Legumes	24.1	Supplements containing energy	5.3
Fish & seafood	18.1	Poultry	2.6 ^a^
Pies & pasties	15.3 ^a^	Pies & pasties	2.6 ^a^
Supplements containing energy	4.6	Red meat	0 ^a^
Vegetarian meat alternatives	0.5 ^a^	Sausages & processed meat	0 ^a^

^a^ Significantly different (*p* < 0.05) proportions of consumers in the self-identified vegetarian and non-vegetarian groups.

**Table 4:** Food group contributions to energy intakes (*n* = 254).

Food Group	Median (25th, 75th Percentile) Energy Intake in Consumers (kJ/Day)		*p*-Value ^a^
	Non-Vegetarians (*n* = 216)	Self-Identified Vegetarians (*n* = 38)	
Bread-based dishes	2325.1 (1440.9, 3075.1)	2044.3 (1312.3, 2352.7)	0.386
Discretionary foods	1436.3 (823.2, 2265.3)	1660.5 (1099.8, 1942.4)	0.963
Vegetarian meat alternatives	519.6	1542.6 (1140.6, 2525.7)	0.181
Pies & pasties	1757.3 (1374.6, 2472.9)	1467.5	0.508
Grains & pasta	1143.0 (726.7, 1732.4)	1167.4 (943.7, 1865.7)	0.304
Bread	973.0 (708.3, 1472.0)	1051.4 (740.4, 1443.4)	0.968
Poultry	815.4 (548.5, 1268.3)	874.3	0.813
Potatoes, kumara, & taro	815.5 (439.2, 1331.9)	851.6 (635.4, 455.3)	0.539
Dairy products	566.2 (270.5, 937.5)	698.5 (218.0, 1469.2)	0.484
Supplements providing energy	416.8 (271.0, 632.0)	680.3 (615.1, 745.5)	0.197
Fish & seafood	611.7 (403.1, 1117.0)	612.6 (558.5, 1152.1)	0.608
Fruit	613.3 (409.9, 957.8)	610.6 (268.5, 875.8)	0.513
Nuts & seeds	521.5 (264.5, 1115.3)	581.5 (281.2, 862.1)	0.990
Eggs & egg dishes	565.0 (328.6, 756.8)	565.0 (565.0, 698.6)	0.634
Legumes	231.4 (118.4, 435.4)	468.2 (343.4, 779.2)	0.011
Cheese	520.5 (295.6, 784.0)	465.1 (244.5, 902.0)	0.750
Breakfast cereals	646.3 (418.0, 1136.7)	452.3 (381.2, 762.3)	0.189
Non-alcoholic beverages	414.8 (155.0, 787.6)	440.5 (143.0, 808.9)	0.960
Milk	378.2 (189.7, 616.1)	367.3 (150.5, 633.1)	0.943
Vegetables	159.0 (79.8, 353.7)	339.0 (127.6, 671.4)	0.012
Fats	259.5 (153.0, 504.4)	245.0 (144.3, 499.3)	0.892
Soups, sauces, & condiments	229.8 (92.1, 462.9)	176.7 (130.7, 497.8)	0.964
Red meat	879.1 (658.3, 1280.2)	0	-
Sausages & processed meat	582.6 (253.2, 1008.2)	0	-

^a^ Differences between vegetarians and non-vegetarians for intake in consumers assessed with a Mann–Whitney test.

**Table 5:** Food group contributions to protein intakes (*n* = 254).

Food Group	Median (25th, 75th Percentile) Protein Intake in Consumers (g/Day)		*p*-Value ^a^
	Non-Vegetarians (*n* = 216)	Self-Identified Vegetarians (*n* = 38)	
Supplements providing energy	10.8 (3.2, 22.5)	25.8 (15.9, 35.7)	0.197
Fish & seafood	19.2 (12.0, 24.5)	19.2 (7.5, 31.0)	0.864
Vegetarian meat alternatives	10.4	18.4 (11.9, 29.5)	0.285
Bread-based dishes	30.2 (14.8, 36.6)	15.5 (10.5, 29.3)	0.118
Eggs & egg dishes	12.6 (6.4, 15.1)	12.6 (12.3, 12.6)	0.883
Poultry	21.6 (14.7, 30.6)	12.0	0.214
Pies & pasties	15.9 (11.4, 21.2)	11.4	0.445
Grains & pasta	8.5 (5.0, 17.4)	8.7 (6.2, 11.1)	0.970
Bread	8.9 (6.0, 12.5)	8.4 (6.4, 10.5)	0.878
Cheese	8.5 (4.9, 12.7)	7.5 (2.0, 17.0)	0.490
Legumes	3.2 (1.6, 7.3)	6.7 (3.2, 10.3)	0.033
Discretionary foods	5.7 (3.1, 8.9)	5.3 (4.6, 8.7)	0.795
Nuts & seeds	5.4 (1.6, 9.0)	4.2 (2.3, 8.7)	0.958
Breakfast cereals	5.0 (3.6, 8.4)	4.2 (3.8, 7.7)	0.869
Vegetables	2.1 (1.0, 4.0)	4.1 (1.9, 6.2)	0.049
Potatoes, kumara, & taro	3.6 (2.0, 5.3)	3.5 (2.2, 5.7)	0.882
Non-alcoholic beverages	1.1 (0.4, 5.6)	2.3 (0.6, 8.2)	0.176
Dairy products	3.5 (1.9, 6.2)	2.2 (1.9, 6.3)	0.308
Soups, sauces, & condiments	0.6 (0.2, 1.9)	2.1 (0.7, 3.6)	0.004
Fruit	1.7 (1.0, 2.6)	1.8 (0.9, 2.4)	0.698
Milk	5.0 (2.1, 8.5)	1.7 (1.1, 8.5)	0.117
Red meat	25.5 (18.0, 37.1)	0	-
Sausages & processed meat	10.1 (5.3, 17.5)	0	-

^a^ Differences between vegetarians and non-vegetarians for intake in consumers assessed with a Mann–Whitney test.

## Data Availability

Data supporting reported results can be obtained, upon reasonable request, from the corresponding author.

## Supplementary Materials

The following supporting information can be downloaded at: https://www.mdpi.com/article/10.3390/nu14030711/s1, Table S1: Food Group Classifications; Table S2: Acceptable macronutrient distribution range (AMDR) of the diets of non-vegetarians and self-identified vegetarians (*n* = 254); Table S3: Food group contributions to fat intakes (*n* = 254); Table S4: Food group contributions to polyunsaturated fat intakes (*n* = 254); Table S5: Food group contributions to saturated fat intakes (*n* = 254); Table S6: Food group contributions to monounsaturated fat intakes (*n* = 254); Table S7: Food group contributions to carbohydrate intakes (*n* = 254); Table S8: Food group contributions to fiber intakes (*n* = 254).
