# Supplementary material for: A Sample of Female Adolescent Self-Identified Vegetarians in New Zealand Consume Less Protein and Saturated Fat, but More Fiber than Their Omnivorous Peers
Source: Nutrients. 2022 Feb 8;14(3):711. doi: 10.3390/nu14030711 (PMC8840649; doi:10.3390/nu14030711)
Supplement: Supplementary file 1 [file nutrients-14-00711-s001.zip › nutrients-1585598-supplementary.pdf]

**Table S1.** Food group classifications.

| Major food group | Minor food groups |
|---|---|
| Grains & pasta | Flour, bran, rye, barley, corn, arrowroot, sago, quinoa, tapioca, semolina, couscous, bulgur, white rice, brown rice, pasta, noodles (including instant), mixed dishes including risotto, sushi, rice-salad, pasta with sauce, lasagne, pasta salad, noodle soup, and other noodle dishes |
| Breakfast cereals | All types including muesli, wheat biscuits, porridge, oats, and puffed/flaked/extruded cereals |
| Bread | All types of break including pita, focaccia, garlic, bagels, crumpets, sweet buns, rolls, tortillas, croutons, pizza base, hot cross buns, papadum, roti and naan. |
| Bread based dishes | Sandwiches, filled rolls, hamburgers, dumplings and pizza with/without meat |
| Discretionary food | Cakes, crisps (chips), biscuits, slices, pancakes, muffins, fruits crumbles, cheesecake, mousse, custard, pudding, creamed rice, sorbet, crackers, chocolate, lollies, sugar, icing, jam, honey, sweet toppings, ice blocks, corn chips, popcorn, grain chips, extruded snacks, muesli bars, fruits bars, puffed cereal bars, nuts and seed bars, bliss balls, snack balls. |
| Milk | All milk, including cow's milk (regular and low fat), soy, almond, rice, cashew, coconut milk for drinking and oat milk |
| Dairy products | Yoghurt, ice cream, cream, sour cream (including vegetarian and vegan alternatives), dairy based dips. |
| Cheese | All types of cheese including edam, colby, cheddar, blue, brie, camembert, feta, haloumi, mozzarella, vegan or vegetarian cheese |
| Fats | Fats, oils, butter, margarine, reduced fat spreads, canola oil, olive oil, sunflower oil, vegetable oil, lard |
| Eggs & egg-based dishes | Poached, boiled, scrambled, and fried eggs, omelettes, egg stir-fries |
| Red meat | Including beef, veal, lamb, mutton, venison and pork (steak, mince, corned beef, roast, schnitzel, chops etc), stir-fries, stews and curries |
| Vegetarian meat alternatives | Tofu, vegan or vegetarian sausages, burger patties, mac'n'cheese, or lasagne. |
| Legumes | All beans and lentils including baked beans, refried beans, chickpeas, hummus, falafel and other bean-based dishes |
| Poultry | Chicken, duck, turkey meat including in stews, stir-fries, curries and chicken nuggets. |
| Processed meat | Sausages, bacon, ham, luncheon, frankfurters, saveloys/cheerios, salami. |
| Pies & pasties | Pies, sausage rolls, quiche with pastry, savouries, pasties |
| Fish & seafood | All fish including fresh, frozen, battered, smoked, canned, fish fingers, shellfish, squid, crab, and other seafood products |
| Vegetables | All vegetables (fresh, frozen, canned) including seaweed, coleslaws, vegetable-based salads with/without meat, and other vegetable dishes |
| Fruits | All fruits canned, cooked, and dried |
| Potatoes, taro & kumara | Mashed boiled and baked potato, hot chips, hash browns, wedges, and other potato dishes |
| Nuts & seeds | Almonds, peanuts, sesame seeds, peanut butter, and other nut butters (including chocolate and nut spreads) nut-based spreads (e.g. pesto) and coconut milk and cream used in cooking |
| Soups, sauces, & condiments | Gravy, tomato and cream-based sauces, soy sauce, mayonnaise, chutney, vinegar based dressings, instant and homemade soups, stocks, and stock powder, marmite, vegemite |
| Non-alcoholic beverages | Fruit/vegetable juice, cordial, soft drinks, powdered drinks, fruit drinks, kombucha, water, sports drinks, energy drinks, milk based beverages including coffee, tea, milk and smoothies |
| Supplements containing energy | Protein powder, protein bars |

**Table S2.** Acceptable macronutrient distribution range (AMDR) of the diets of non-vegetarians and self-identified vegetarians ($n$ = 254).

| | **Non-vegetarians ($n$ = 216)** | | | **Self-identified vegetarians ($n$ = 38)** | | | |
|---|---|---|---|---|---|---|---|
| | **Below AMDR $n$ (%)** | **Above AMDR $n$ (%)** | **Within AMDR $n$ (%)** | **Below AMDR $n$ (%)** | **Above AMDR $n$ (%)** | **Within AMDR $n$ (%)** | **$p$-value[a]** |
| Fat between 20–35 % kJ | 3 (1.4) | 159 (73.6) | 54 (25.0) | 0 | 27 (71.1) | 11 (29.0) | 0.607 |
| Saturated fat less than 10% kJ | n/a | 203 (94.0) | 13 (6.0) | n/a | 29 (76.3) | 9 (23.7) | <0.001 |
| Protein between 15–25 % kJ | 93 (43.1) | 0 | 123 (56.9) | 28 (73.7) | 0 | 10 (26.3) | <0.001 |
| Carbohydrate between 45–65 % kJ | 72 (33.3) | 0 | 144 (66.7) | 11 (28.9) | 0 | 27 (71.1) | 0.595 |

[a] $p$-value for the difference between non-vegetarians and self-identified vegetarians in the proportions of those within the AMDR, using a chi-squared test.

**Table S3.** Food group contributions to fat intakes ($n$ = 254).

| Food group | Median (25th, 75th percentile) fat intake in consumers (g/day) | | $p$-value[a] |
|---|---|---|---|
| | Non-vegetarians ($n$ = 216) | Self-identified vegetarians ($n$ = 38) | |
| Bread-based dishes | 21.1 (12.3, 33.1) | 18.2 (8.9, 32.0) | 0.647 |
| Discretionary foods | 14.9 (9.0, 24.4) | 17.1 (10.4, 28.7) | 0.494 |
| Vegetarian meat alternatives | 0.7 | 15.1 (11.5, 29.7) | 0.109 |
| Poultry | 9.6 (5.2, 17.4) | 15.0 | 0.496 |
| Pies & pasties | 22.7 (18.7, 38.9) | 13.8 | 0.203 |
| Nuts & seeds | 10.5 (4.9, 22.6) | 10.7 (6.2, 18.4) | 0.978 |
| Dairy products | 6.0 (2.2, 12.1) | 10.0 (3.3, 19.9) | 0.101 |
| Eggs & egg dishes | 9.4 (5.7, 13.9) | 9.4 (9.4, 13.3) | 0.611 |
| Cheese | 10.0 (5.5, 16.2) | 8.9 (4.8, 15.9) | 0.773 |
| Fish & seafood | 7.4 (1.9, 13.5) | 8.1 (7.9, 14.4) | 0.542 |
| Potatoes, kumara, & taro | 6.3 (2.6, 13.4) | 7.6 (5.8, 13.5) | 0.547 |
| Fats | 7.0 (4.1, 13.6) | 6.6 (3.9, 13.5) | 0.892 |
| Grains & pasta | 3.5 (0.8, 10.7) | 4.5 (1.0, 9.1) | 0.864 |
| Milk | 3.8 (1.9, 6.4) | 3.8 (1.4, 8.0) | 0.615 |
| Bread | 3.2 (1.8, 5.7) | 3.7 (1.5, 6.1) | 0.729 |
| Supplements providing energy | 1.5 (0.6, 2.3) | 3.3 (2.9, 3.7) | 0.197 |
| Legumes | 1.5 (0.4, 2.9) | 3.0 (2.1, 7.6) | 0.012 |
| Vegetables | 0.6 (0.3, 3.3) | 1.5 (0.5, 6.0) | 0.013 |
| Soups, sauces, & condiments | 3.5 (0.1, 9.1) | 0.8 (0.1, 5.7) | 0.181 |
| Breakfast cereals | 1.4 (0.7, 4.4) | 0.7 (0.6, 2.3) | 0.147 |
| Fruit | 1.0 (0.5, 1.7) | 0.7 (0.5, 2.6) | 0.391 |
| Non-alcoholic beverages | 0.5 (0.1, 0.3) | 0.6 (0.2, 4.1) | 0.246 |
| Red meat | 11.1 (7.7, 16.9) | 0 | - |
| Sausages & processed meat | 10.1 (3.4, 19.1) | 0 | - |

[a] Differences between vegetarians and non-vegetarians for intake in consumers assessed with a Mann-Whitney test.

**Table S4.** Food group contributions to polyunsaturated fat intakes ($n$ = 254).

| Food group | Median (25th, 75th percentile) polyunsaturated fat intake in consumers (g/day) | | $p$-value[a] |
|---|---|---|---|
| | Non-vegetarians ($n$ = 216) | Self-identified vegetarians ($n$ = 38) | |
| Vegetarian meat alternatives | 0.4 | 5.8 (2.8, 12.8) | 0.109 |
| Fish & seafood | 1.1 (0.4, 2.8) | 4.1 (2.0, 4.3) | 0.060 |
| Discretionary foods | 2.0 (0.9, 3.8) | 3.2 (1.8, 5.1) | 0.051 |
| Nuts & seeds | 2.7 (1.2, 6.2) | 2.5 (1.2, 4.3) | 0.725 |
| Pies & pasties | 1.1 (0.7, 2.5) | 2.5 | 0.386 |
| Bread-based dishes | 2.5 (1.4, 3.9) | 1.9 (0.7, 4.3) | 0.508 |
| Poultry | 1.3 (0.6, 2.5) | 1.7 | 0.636 |
| Fats | 0.8 (0.4, 1.7) | 1.6 (0.4, 2.4) | 0.254 |
| Potatoes, kumara, & taro | 1.0 (0.3, 2.1) | 1.3 (0.4, 3.6) | 0.480 |
| Legumes | 0.6 (0.2, 1.2) | 1.2 (0.4, 1.8) | 0.032 |
| Eggs & egg dishes | 1.0 (0.6, 1.6) | 1.0 (1.0, 1.1) | 0.831 |
| Bread | 0.9 (0.5, 1.6) | 1.0 (0.4, 1.7) | 0.992 |
| Grains & pasta | 0.6 (0.3, 1.4) | 0.7 (0.3, 1.8) | 0.223 |
| Supplements providing energy | 0.5 (0.1, 0.8) | 0.7 (0.1, 1.3) | 0.667 |
| Soups, sauces, & condiments | 1.0 (0.2, 2.5) | 0.4 (0.1, 1.6) | 0.071 |
| Dairy products | 0.2 (0.1, 0.3) | 0.4 (0.1, 1.3) | 0.011 |
| Milk | 0.2 (0.1, 0.3) | 0.4 (0.2, 1.1) | <0.001 |
| Breakfast cereals | 0.6 (0.3, 1.5) | 0.3 (0.2, 1.0) | 0.157 |
| Vegetables | 0.2 (0.1, 0.6) | 0.3 (0.2, 1.9) | 0.105 |
| Fruit | 0.3 (0.1, 0.6) | 0.2 (0.1, 0.4) | 0.126 |
| Cheese | 0.3 (0.1, 0.4) | 0.2 (0.1, 0.5) | 0.688 |
| Non-alcoholic beverages | 0.0 (0.0, 0.2) | 0.1 (0.0, 0.4) | 0.228 |
| Sausages & processed meat | 0.7 (0.3, 1.4) | - | - |
| Red meat | 0.6 (0.3, 1.3) | - | - |

[a] Differences between vegetarians and non-vegetarians for intake in consumers assessed with a Mann-Whitney test.

**Table S5.** Food group contributions to saturated fat intakes ($n$ = 254).

| Food group | Median (25th, 75th percentile) saturated fat intake in consumers (g/day) | | $p$-value[a] |
|---|---|---|---|
| | Non-vegetarians ($n$ = 216) | Self-identified vegetarians ($n$ = 38) | |
| Bread-based dishes | 9.0 (5.1, 14.5) | 7.9 (2.9, 17.2) | 0.971 |
| Poultry | 2.8 (1.6, 5.2) | 6.6 | 0.261 |
| Cheese | 6.5 (3.6, 10.2) | 6.5 (2.7, 10.3) | 0.808 |
| Dairy products | 3.9 (1.4, 8.0) | 6.2 (2.2, 13.0) | 0.154 |
| Discretionary foods | 6.6 (3.9, 11.3) | 6.0 (4.4, 11.9) | 0.724 |
| Pies & pasties | 11.8 (9.9, 17.4) | 5.3 | 0.139 |
| Vegetarian meat alternatives | 0.1 | 3.8 (1.7, 6.4) | 0.109 |
| Eggs & egg dishes | 2.7 (1.6, 4.1) | 2.7 (2.7, 4.5) | 0.460 |
| Potatoes, kumara, & taro | 1.7 (0.5, 3.9) | 2.7 (1.0, 3.8) | 0.528 |
| Fats | 2.3 (1.0, 4.7) | 1.8 (1.3, 4.1) | 0.957 |
| Nuts & seeds | 1.6 (0.8, 3.0) | 1.7 (0.7, 3.7) | 0.728 |
| Supplements providing energy | 0.7 (0.1, 1.1) | 1.6 (1.4, 1.9) | 0.099 |
| Fish & seafood | 1.2 (0.4, 2.5) | 1.1 (1.0, 2.2) | 0.903 |
| Grains & pasta | 0.9 (0.2, 3.7) | 0.8 (0.3, 2.3) | 0.913 |
| Legumes | 0.2 (0.1, 0.5) | 0.6 (0.3, 0.9) | 0.010 |
| Milk | 1.7 (0.6, 3.8) | 0.5 (0.2, 3.1) | 0.020 |
| Bread | 0.4 (0.2, 1.0) | 0.4 (0.3, 0.8) | 0.836 |
| Non-alcoholic beverages | 0.2 (0.0, 0.6) | 0.3 (0.1, 0.7) | 0.189 |
| Vegetables | 0.1 (0.0, 0.6) | 0.3 (0.1, 1.2) | 0.027 |
| Soups, sauces, & condiments | 0.9 (0.1, 2.3) | 0.2 (0.0, 1.3) | 0.079 |
| Fruit | 0.2 (0.1, 0.4) | 0.2 (0.0, 0.4) | 0.401 |
| Breakfast cereals | 0.2 (0.1, 0.9) | 0.2 (0.1, 0.3) | 0.565 |
| Red meat | 4.7 (3.0, 7.6) | - | - |
| Sausages & processed meat | 3.7 (1.3, 7.7) | - | - |

[a] Differences between vegetarians and non-vegetarians for intake in consumers assessed with a Mann-Whitney test.

**Table S6.** Food group contributions to monounsaturated fat intakes ($n$ = 254).

| Food group | Median (25th, 75th percentile) monounsaturated fat intake in consumers (g/day) | | $p$-value[a] |
|---|---|---|---|
| | Non-vegetarians ($n$ = 216) | Self-identified vegetarians ($n$ = 38) | |
| Bread-based dishes | 7.8 (3.9, 12.0) | 6.9 (1.6, 10.1) | 0.348 |
| Discretionary foods | 4.7 (2.5, 8.2) | 5.3 (3.2, 9.5) | 0.357 |
| Poultry | 4.5 (2.5, 7.6) | 5.2 | 0.836 |
| Vegetarian meat alternatives | 0.1 | 5.1 (2.7, 9.1) | 0.109 |
| Pies & pasties | 7.7 (6.0, 12.8) | 5.0 | 0.285 |
| Fish & seafood | 2.6 (0.4, 5.8) | 4.7 (1.7, 6.8) | 0.479 |
| Nuts & seeds | 5.3 (2.4, 10.1) | 4.3 (3.5, 11.7) | 0.732 |
| Eggs & egg dishes | 3.5 (2.2, 5.2) | 3.8 (3.8, 5.0) | 0.460 |
| Potatoes, kumara, & taro | 3.0 (1.0, 6.8) | 3.0 (2.4, 6.4) | 0.606 |
| Fats | 2.8 (1.4, 5.2) | 2.9 (1.0, 5.1) | 0.996 |
| Dairy products | 1.3 (0.6, 2.8) | 2.9 (0.7, 4.0) | 0.145 |
| Cheese | 2.2 (1.2, 3.5) | 2.1 (0.8, 3.0) | 0.467 |
| Milk | 1.0 (0.5, 1.9) | 1.9 (0.8, 2.1) | 0.072 |
| Legumes | 0.5 (0.0, 1.2) | 1.4 (0.9, 2.1) | 0.027 |
| Bread | 0.9 (0.4, 1.7) | 1.3 (0.4, 2.1) | 0.600 |
| Grains & pasta | 0.9 (0.2, 3.7) | 1.1 (0.3, 2.7) | 0.750 |
| Soups, sauces, & condiments | 1.9 (0.4, 4.7) | 0.9 (0.1, 2.8) | 0.169 |
| Supplements providing energy | 0.3 (0.2, 1.4) | 0.6 (0.5, 0.7) | 0.519 |
| Vegetables | 0.1 (0.0, 1.0) | 0.6 (0.1, 3.2) | 0.006 |
| Non-alcoholic beverages | 0.1 (0.0, 0.7) | 0.2 (0.1, 1.4) | 0.158 |
| Breakfast cereals | 0.3 (0.1, 1.5) | 0.1 (0.1, 0.3) | 0.594 |
| Fruit | 0.1 (0.0, 0.2) | 0.1 (0.0, 1.1) | 0.684 |
| Red meat | 4.6 (2.9, 7.0) | - | - |
| Sausages & processed meat | 4.3 (1.3, 8.1) | - | - |

[a] Differences between vegetarians and non-vegetarians for intake in consumers assessed with a Mann-Whitney test.

**Table S7.** Food group contributions to carbohydrate intakes ($n$ = 254).

| Food group | Median (25th, 75th percentile) carbohydrate intake in consumers (g/day) | | $p$-value[a] |
|---|---|---|---|
| | Non-vegetarians ($n$ = 216) | Self-identified vegetarians ($n$ = 38) | |
| Discretionary foods | 48.4 (26.6, 76.3) | 51.5 (30.2, 62.5) | 0.638 |
| Grains & pasta | 41.0 (27.1, 59.3) | 47.0 (37.5, 81.3) | 0.033 |
| Vegetarian meat alternatives | 19.2 | 46.3 (13.0, 53.9) | 0.593 |
| Pies & pasties | 30.8 (21.1, 41.8) | 45.9 | 0.208 |
| Bread | 41.6 (29.7, 64.8) | 42.1 (28.1, 58.8) | 0.766 |
| Bread-based dishes | 50.2 (34.3, 84.3) | 41.3 (37.0, 62.8) | 0.605 |
| Potatoes, kumara, & taro | 29.4 (15.6, 42.8) | 33.2 (18.7, 49.2) | 0.296 |
| Fruit | 29.6 (19.4, 48.4) | 28.7 (11.9, 37.0) | 0.114 |
| Non-alcoholic beverages | 20.6 (8.9, 39.9) | 20.6 (5.7, 31.0) | 0.399 |
| Breakfast cereals | 28.4 (18.5, 47.6) | 19.8 (17.5, 34.0) | 0.259 |
| Legumes | 6.1 (3.0, 15.6) | 11.9 (5.0, 26.4) | 0.092 |
| Milk | 8.5 (3.7, 11.7) | 9.4 (4.9, 12.8) | 0.357 |
| Supplements providing energy | 3.3 (2.1, 13.0) | 7.6 (2.5, 12.7) | 0.830 |
| Poultry | 8.0 (2.1, 14.8) | 7.1 | 0.838 |
| Dairy products | 14.0 (3.9, 25.6) | 7.0 (2.3, 30.1) | 0.703 |
| Fish & seafood | 8.4 (4.2, 14.0) | 7.0 (6.1, 7.9) | 0.688 |
| Vegetables | 5.0 (2.5, 11.2) | 7.0 (3.8, 23.0) | 0.028 |
| Soups, sauces, & condiments | 3.1 (0.8, 8.0) | 6.8 (2.6, 13.2) | 0.007 |
| Nuts & seeds | 2.4 (0.9, 6.4) | 1.9 (1.1, 4.8) | 0.678 |
| Eggs & egg dishes | 0.3 (0.2, 0.6) | 0.5 (0.0, 0.5) | 0.858 |
| Cheese | 0.1 (0.0, 0.4) | 0.3 (0.0, 4.2) | 0.555 |
| Red meat | 6.6 (0.5, 10.3) | 0 | - |
| Sausages & processed meat | 1.1 (0.4, 5.9) | 0 | - |

[a] Differences between vegetarians and non-vegetarians for intake in consumers assessed with a Mann-Whitney test.

**Table S8.** Food group contributions to fibre intakes ($n$ = 254).

| Food group | Median (25th, 75th percentile) fibre intake in consumers (g/day) | | $p$-value[a] |
|---|---|---|---|
| | Non-vegetarians ($n$ = 216) | Self-identified vegetarians ($n$ = 38) | |
| Vegetarian meat alternatives | 9.2 | 7.8 (4.5, 13.3) | 0.789 |
| Legumes | 3.2 (1.9, 7.1) | 5.1 (2.2, 10.1) | 0.321 |
| Bread-based dishes | 4.5 (2.8, 8.5) | 4.7 (2.7, 8.8) | 0.536 |
| Vegetables | 3.0 (1.6, 5.3) | 4.5 (2.0, 6.7) | 0.123 |
| Bread | 4.3 (3.0, 6.4) | 4.3 (2.9, 5.5) | 0.508 |
| Supplements providing energy | 0.7 (0.1, 2.7) | 4.3 | 0.134 |
| Fruit | 5.1 (3.1, 7.6) | 4.0 (2.4, 6.1) | 0.116 |
| Potatoes, kumara, & taro | 3.4 (1.9, 5.0) | 3.7 (2.7, 6.3) | 0.270 |
| Grains & pasta | 2.5 (1.4, 4.3) | 3.6 (2.4, 5.4) | 0.035 |
| Breakfast cereals | 3.4 (1.9, 5.8) | 3.2 (2.3, 6.4) | 0.934 |
| Discretionary foods | 2.6 (1.2, 4.7) | 3.2 (1.7, 5.0) | 0.476 |
| Pies & pasties | 2.0 (1.3, 3.3) | 3.1 | 0.462 |
| Nuts & seeds | 2.0 (0.6, 3.5) | 1.8 (0.8, 3.7) | 0.695 |
| Fish & seafood | 0.6 (0.3, 1.1) | 1.2 (0.4, 2.0) | 0.531 |
| Poultry | 1.1 (0.5, 2.1) | 1.2 | 0.956 |
| Soups, sauces, & condiments | 0.4 (0.2, 1.0) | 0.8 (0.4, 2.3) | 0.060 |
| Non-alcoholic beverages | 0.5 (0.2, 1.3) | 0.7 (0.3, 1.8) | 0.379 |
| Milk | 0.5 (0.4, 1.0) | 0.6 (0.3, 1.1) | 0.844 |
| Dairy products | 0.3 (0.2, 0.5) | 0.4 (0.2, 2.1) | 0.299 |
| Sausages & processed meat | 1.7 (0.6, 2.5) | - | - |
| Red meat | 1.2 (0.4, 2.4) | - | - |

[a] Differences between vegetarians and non-vegetarians for intake in consumers assessed with a Mann-Whitney test.
